# The presence, clarity, and consistency of definitions in pregnancy outcomes in infertility trials: a systematic review

**DOI:** 10.1093/humrep/deaf022

**Published:** 2025-02-21

**Authors:** Qian Feng, Wentao Li, Wanlin Li, Rui Wang, James Crispin, Salvatore Longobardi, Thomas D’Hooghe, Ben W Mol

**Affiliations:** Department of Obstetrics and Gynaecology, Monash University, Melbourne, Victoria, Australia; Department of Obstetrics and Gynaecology, Monash University, Melbourne, Victoria, Australia; National Perinatal Epidemiology and Statistics Unit (NPESU), Centre for Big Data Research in Health, University of New South Wales, Sydney, NSW, Australia; Faculty of Medicine, School of Clinical Medicine, University of New South Wales, Sydney, NSW, Australia; Department of Obstetrics and Gynaecology, Monash University, Melbourne, Victoria, Australia; Department of Obstetrics and Gynaecology, Monash University, Melbourne, Victoria, Australia; NHMRC Clinical Trials Centre, University of Sydney, Sydney, NSW, Australia; Department of Obstetrics and Gynaecology, Monash University, Melbourne, Victoria, Australia; Global Clinical Development Fertility, Research and Development, Merck, Darmstadt, Germany; Research Group Reproductive Medicine, Department of Development and Regeneration, Organ Systems, Group Biomedical Sciences, KU Leuven (University of Leuven), Leuven, Belgium; Department of Obstetrics, Gynecology and Reproductive Sciences, Yale School of Medicine, New Haven, CT, USA; Global Medical Affairs Fertility, Research and Development, Merck Healthcare KGaA, Darmstadt, Germany; Department of Obstetrics and Gynaecology, Monash University, Melbourne, Victoria, Australia; Aberdeen Centre for Women’s Health Research, School of Medicine, Medical Sciences and Nutrition, University of Aberdeen, Aberdeen, UK

**Keywords:** infertility, quality of reporting, randomized controlled trial, research quality, IVF/ICSI outcomes, live birth, pregnancy, ongoing pregnancy, clinical pregnancy

## Abstract

**STUDY QUESTION:**

How frequently do infertility trials report live birth and pregnancy, and how consistently were their definitions reported?

**SUMMARY ANSWER:**

One-third of 1425 infertility trials published in the last decade reported live birth, with one in eight reporting clinical pregnancy, ongoing pregnancy, and live birth concurrently; absent, ambiguous, or heterogeneous definitions were common.

**WHAT IS KNOWN ALREADY:**

Absent or inconsistent outcome definitions in randomized controlled trials (RCTs) limit their interpretation and complicate subsequent evidence synthesis. While reporting live birth in infertility trials has been a long-running recommendation, the extent to which this is adhered to, and the temporal trend of adherence, is unclear. Furthermore, it is unknown if outcome reporting in infertility trials is clear and consistent.

**STUDY DESIGN, SIZE, DURATION:**

We studied all RCTs in infertility published between 2012 and 2023. We aimed to assess (i) whether biochemical pregnancy, clinical pregnancy, ongoing pregnancy, and live birth were reported; the temporal trends in reporting these pregnancy outcomes, and compare the characteristics of trials reporting each type of outcome; (ii) whether and how these pregnancy outcomes were defined.

**PARTICIPANTS/MATERIALS, SETTING, METHODS:**

We systematically searched Embase, Medline, and CENTRAL for RCTs in infertility from January 2012 to August 2023. RCTs involving infertile women that reported either biochemical pregnancy, clinical pregnancy, ongoing pregnancy, or live birth were eligible. Secondary analyses, interim analyses, or conference abstracts were not eligible. Two authors independently screened articles. We extracted pregnancy definitions and trial characteristics primarily using text mining in R, a programming environment for data analysis, and supplemented by manual checking. The accuracy of extracted data was validated in a random sample of 50 articles, with sensitivity and specificity all at or above 90%.

**MAIN RESULTS AND THE ROLE OF CHANCE:**

We included 1425 infertility RCTs. Among these, 419 (29.4%) reported biochemical pregnancy. While 1359 (95.4%) RCTs reported clinical pregnancy, 404 (28.4%) reported ongoing pregnancy, and 484 (34.0%) reported live birth, only 174 (12.2%) reported all three outcomes. The proportion of trials reporting live birth increased from 23.1% in 2012 to 33.7% in 2023. Trials reporting up to biochemical pregnancy or clinical pregnancy were more likely to be unregistered, smaller, single-centered, and published in non-first quarter journals. Definitions for biochemical, clinical, ongoing pregnancy, and live birth were provided in 68.5% (287/419), 64.5% (876/1359), 70.5% (285/404), and 41.1% (199/484) of articles reporting on these outcomes. Among 876 clinical pregnancy definitions, 63.4% (n = 555) specified the pregnancy confirmation timing. Of the 220 definitions that reported gestational weeks (ranging from 4 to 16 weeks), the most common cut-off was 6 weeks, used in 48.2% (n = 106) of cases. For ongoing pregnancy definitions, 96.1% (n = 274) of the 285 definitions included gestational age in weeks (ranging from 6 to 32 weeks), with 12 weeks being the most common cut-off used in 49.1% (n = 140) of definitions. Among 199 live birth definitions, 62.3% (n = 124) used a gestational age threshold (ranging from 20 to 37 weeks), with 24 weeks being the most common cut-off, used in 28.6% (n = 57) of trials.

**LIMITATIONS, REASONS FOR CAUTION:**

Due to the vast data we needed to extract, we used text-mining supplemented by manual data extraction. While we optimized the text-mining algorithm attempting to identify all types of outcome definitions and manually curated all extracted definitions, definitions were missed in less than 10% of randomly checked studies, which is a limitation of this study. We only described definition patterns in published RCTs, and our results cannot be extrapolated to unpublished RCTs.

**WIDER IMPLICATIONS OF THE FINDINGS:**

Despite long-standing recommendations to report live birth in infertility trials, in the last decade only a third of RCTs did so. This highlights a disconnection between the advocated outcome and what researchers are reporting. We observed an encouraging trend that there has been a consistent rise in the proportion of trials reporting live birth. Furthermore, the significant lack and variability of pregnancy definitions underscore the imperative to increase the dissemination and uptake of standardized pregnancy outcomes.

**STUDY FUNDING/COMPETING INTEREST(S):**

No funding was received for the study. Q.F. reports receiving a PhD scholarship from Merck. B.W.M. is supported by an NHMRC Investigator grant (GNT1176437). B.W.M. reports consultancy, travel support, and research funding from Merck and consultancy for Organon and Norgine. B.W.M. holds stock from ObsEva. W.T.L. is supported by an NHMRC Investigator grant (GTN2016729). W.L.L. reports receiving a PhD scholarship from the China Scholarship Council. T.D.H and S.L. are employees of Merck Healthcare KGaA, Darmstadt, Germany. R.W. is supported by an NHMRC Investigator grant (GTN2009767). The other author has no conflict of interest to declare.

**REGISTRATION NUMBER:**

CRD42024498624.

## Introduction

Infertility is defined as the inability to conceive after 12 months of regular, unprotected sexual intercourse (Zegers-Hochschild *et al.*, 2017a, b). In the last few decades, ground-breaking innovations such as IVF have revolutionized reproductive medicine, thus giving millions of infertile couples a path to parenthood. Such achievements are remarkable in a world where one in every six couples suffers from infertility (World Health Organization 2023). To achieve a live birth, infertile couples often need to go through more than one treatment cycle. In 2021, 20 thousand babies were born in Australia after ART, which involved the initiation of 111 thousand cycles, highlighting the importance of identifying safe and effective treatments to streamline this treatment journey (Newman *et al.*, 2023).

**Figure 1.**
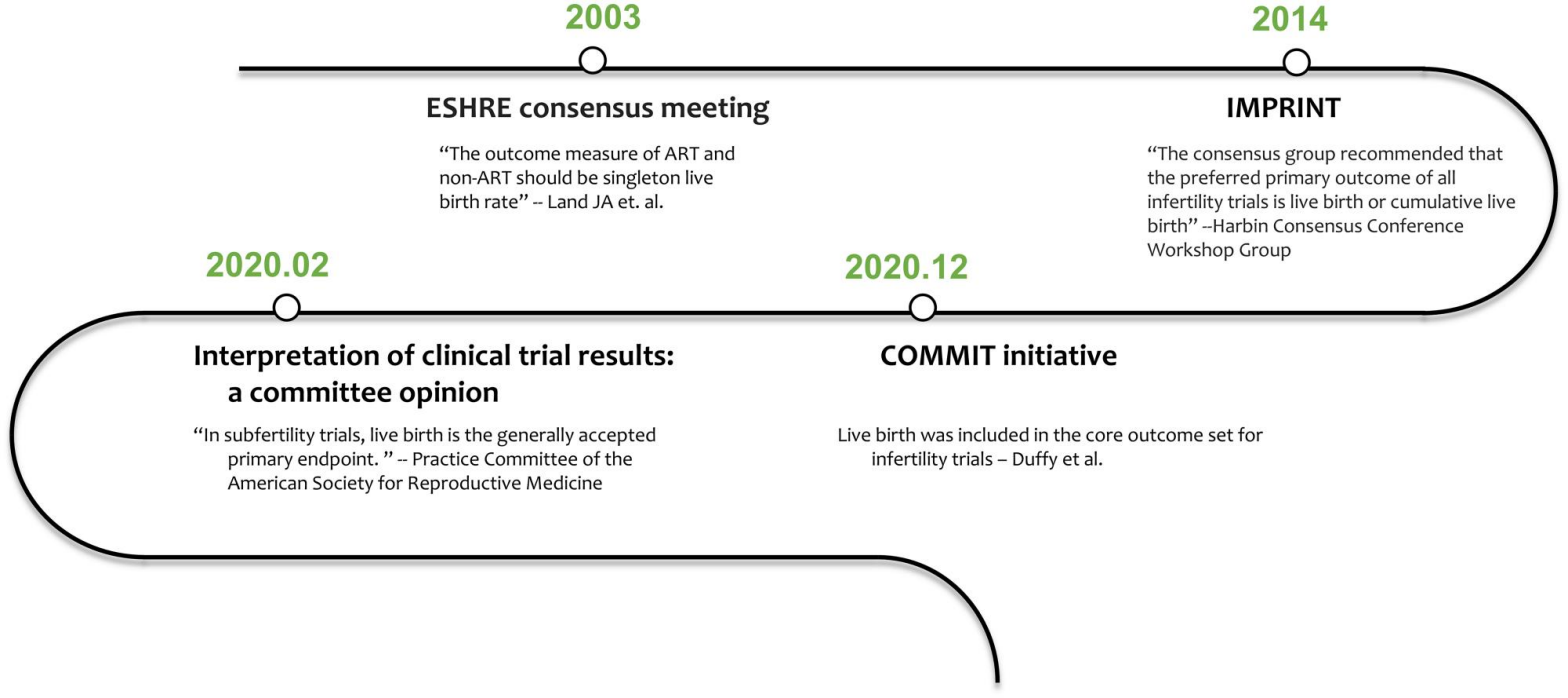
**Recommendations of reporting live birth in infertility trials from professional bodies or expert meetings.** IMPRINT, Improving the Reporting of Clinical Trials of Infertility Treatments; COMMIT, Core Outcome Measure for Infertility Trials. For the searching method, we employed a snowballing search, beginning with the study by Land and Evers (2003), to identify guidelines or consensus statements that recommended reporting live births.

High-quality randomized controlled trials (RCTs) stand as a pivotal solution to the challenge of producing evidence for the effectiveness of treatments. Despite the proliferation of RCTs in quantity, there is still room for improvement in methodological rigor. One area for enhancement is ensuring that the outcomes of trials are pertinent to patients (Chalmers and Glasziou, 2009). In response, professional bodies such as ESHRE have recommended that all RCTs in reproductive medicine report live birth (Land and Evers, 2003; Barnhart, 2014; Practice Committee of the ASRM 2020) (Fig. 1). Live birth has also been prioritized in the core outcome sets (COS), a consensus-based standardized collection of outcomes that should be measured and reported as a minimum in clinical trials (Duffy *et al.*, 2020a, b, 2021). Despite the long-standing recommendation, the extent to which this is adhered to is unclear.

Additionally, the absent, inconsistent, or unclear definitions of outcomes are also to be improved in the reporting of RCTs; these issues contribute to ambiguity or exaggeration of the treatment effects, heterogeneity in evidence synthesis, and barriers to extrapolating results to other populations. A review of RCTs in reproductive medicine in 2016 revealed considerable variability in how pregnancy outcomes were defined, with half of the live birth definitions missing; among those defined, the gestational weeks for live birth ranged from 24 to 28 or later (Wilkinson *et al.*, 2016). To protect trials against this methodological frailty, standardized outcomes by The International Committee for Monitoring Assisted Reproductive Technologies (ICMART) were developed in 2009 and updated in 2017 (Zegers-Hochschild *et al.*, 2009, 2017a, b), both of which were endorsed by ESHRE and ASRM. Furthermore, standardized definitions were also included in COS (Duffy *et al.*, 2020a, b) (Supplementary Table S1). However, it remains unknown whether the heterogeneity and the extent of absence of outcome definitions have abated since these initiatives. Bridging this research gap is crucial to avoid the standardized definitions being developed but rarely used.

Here, we aim to (i) assess how many trials had reported biochemical pregnancy, clinical pregnancy, ongoing pregnancy, and live birth, and compare the characteristics between trials that reported different endpoints; (ii) evaluate the variability in pregnancy definitions, including biochemical, clinical, ongoing pregnancy, and live birth.

## Materials and methods

We performed a systematic review involving RCTs published in Infertility from January 2012 to August 2023. The study was registered in PROSPERO (CRD42024498624) and the protocol was uploaded to OSF (https://osf.io/n4r7m/) before the study started.

### Literature search

We searched Medline, Embase, and Cochrane Central Register of Controlled Trials (CENTRAL) for RCTs in infertility published from 1 January 2012 to 30 August 2023 using a comprehensive search strategy (Supplementary Data File S1). We included RCTs that had randomized women or couples with infertility and reported biochemical pregnancy, clinical pregnancy, ongoing pregnancy, and pregnancy that did not specify the stage or live birth. We excluded secondary analyses, interim analyses, or conference abstracts. Two independent reviewers (W.L.L. and J.C.) screened the articles and the discrepancies were resolved through discussions with the first author (Q.F.). Articles written in English or other languages were all included.

To ensure our search was comprehensive, we developed a test set of articles that we independently searched in the Cochrane Library. A detailed description of the development method is in Supplementary Data File S2. In brief, we searched for Cochrane reviews published from 2013 to 2023 and manually selected trials cited by these reviews that had reported pregnancy or live birth.

### Data parsing and extractions

We downloaded all articles in portable document format (PDF) via Endnote ‘find full text’ functions or manually through university subscriptions or interlibrary loans. These PDFs were then converted into Extensible Markup Language files, a format that allows computers to read and process the content. This conversion was carried out using Grobid, a machine-learning software specifically designed to parse academic articles (Lopez, 2009). The reliability of Grobid for parsing data outperforms other software (Tkaczyk *et al.*, 2018; Foppiano *et al.*, 2023).

Our data extraction for English-written articles primarily relied on text-mining, a process in which computers recognize and extract information from human natural languages. This automated process was complemented by manual extraction to ensure accuracy and thoroughness. This semi-automated data extraction has been widely used by authors of meta-research for extracting data from a large number of articles (Chavalarias and Ioannidis, 2010; Lajeunesse and Fitzjohn, 2016; Zavalis *et al.*, 2023). This process began with the development of algorithms designed to recognize variations of expressions that are relevant to pregnancy definitions and trial characteristics, including trial registration, sample size, and patient recruitment country. We then tested the performance of the algorithm by comparing the data extracted by the algorithm and those extracted by a human from 30 randomly selected articles. The algorithm was then iteratively tinkered until 90% of the targeted data were successfully extracted. Once the algorithm was built, all full articles were subjected to automatic data extraction. The manual data extraction method was used when the data could not be successfully extracted using machine learning. For example, this occurred when sample sizes were missing or when the extracted data seemed implausible, such as when clinical pregnancy was defined as 24 weeks of gestation.

To ensure the above semi-automated data extraction method was reliable, we randomly searched 50 articles from all included articles to manually check the results. Given the absence of a consensus over the percentage of articles required for validation, we selected our sample size to balance the practical feasibility of manual checking with the need to test the algorithm’s reliability.

If an article stated that the outcomes were defined as previously published or the protocol, we searched the cited references and manually extracted the full definitions. Articles written in a language other than English were translated using Google Translate and then the data were extracted manually or with the help of a native speaker.

### Other data sources

The list of first-quarter journals was retrieved from Clarivate’s Journal Citation Reports published in 2023 (Clarivate’s, 2023). The funding source and citation times were obtained from Dimensions, a large literature database chosen for its reliable and comprehensive meta-data (Dimensions, 2024).

### Statistical analysis

Proportions and frequencies were used to describe the trend. All data extraction, statistical analyses, and graphical representations were conducted using R software (R Core Team 2013).

## Results

**Figure 2.**
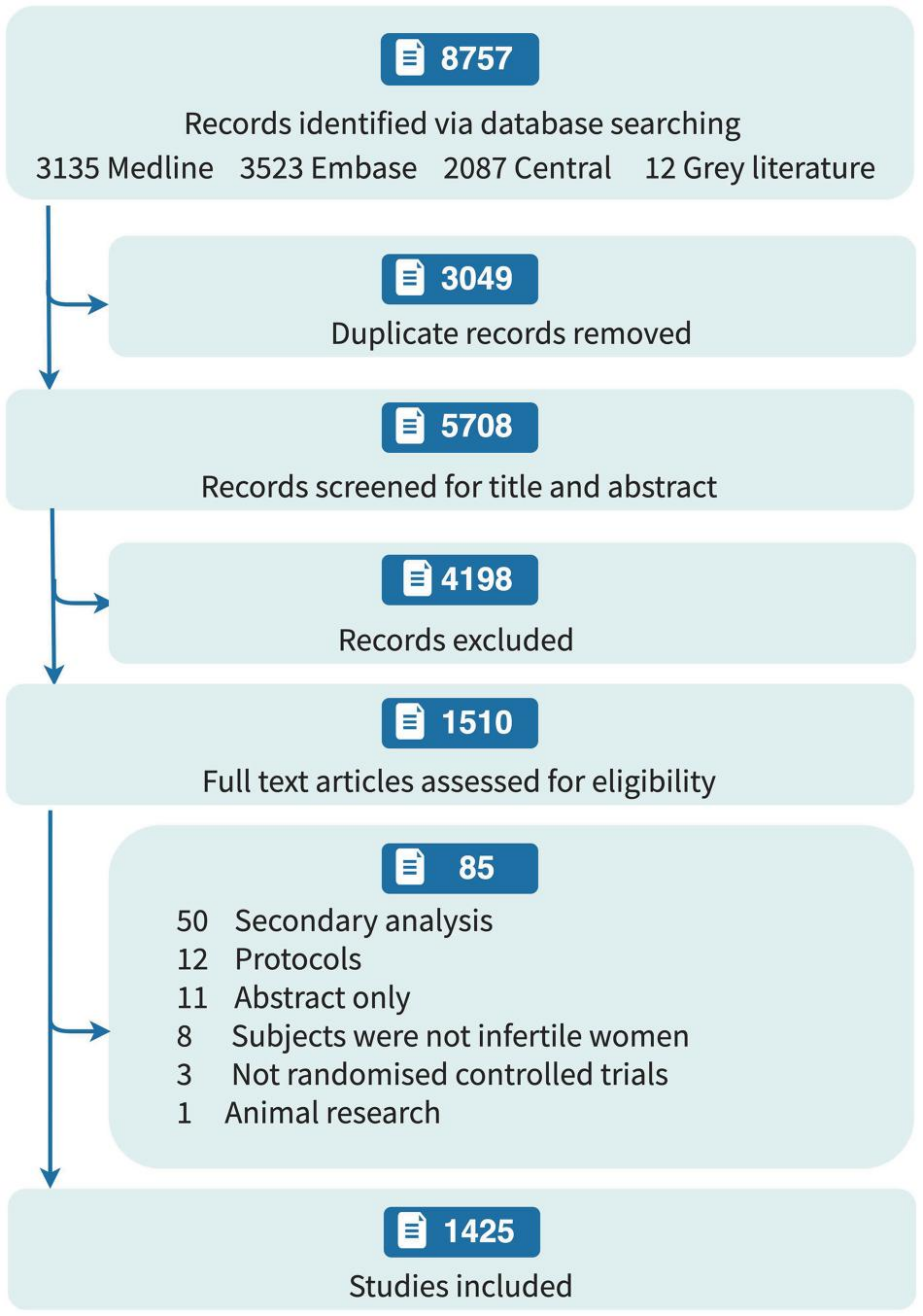
**Flowchart of the study**.

From 8757 records detected, 1510 RCTs were found eligible after manually perusing the title and abstract (Fig. 2). Upon full article review, an additional 85 articles were removed as they were secondary analyses, protocols, conference abstracts, or non-RCTs, leaving 1425 RCTs for our analysis. These 1425 RCTs were then compared against the 84 RCTs in the test set, from which we successfully identified 77 in our search results, representing 91.7% of the RCTs in the test set.

### Manual checking data extraction

To ensure our semi-automated data extraction method is valid, we conducted a manual check to confirm the accuracy of all extracted data. Our manual checking was composed of three parts, data extraction of trial characteristics, pregnancy outcome reporting ascertainment and extraction of pregnancy definition. On a manual checking of 50 articles for the accuracy of extracting all extracted data, the sensitivity was 92% for each type of data extracted and the specificity was 100%. The detailed breakdown of manual checking results by data type can be found in Supplementary Data File S3.

In the 50 articles, 47 reported clinical pregnancy, 12 reported ongoing pregnancy, and 15 reported live birth. This manual checking revealed that our data extraction for ascertaining outcome reporting had missed nine endpoints, with a sensitivity of 89.2% and a specificity of 98.6%. A breakdown of errors in each pregnancy endpoint is provided in Supplementary Data File S4.

The 50 randomly selected articles contained 44 definitions, including six biochemical pregnancy definitions, 23 clinical pregnancy definitions, 10 ongoing pregnancy definitions, and five live birth definitions. Our automated data extraction of these definitions had missed four definitions when extracting these 44 definitions, with a sensitivity of 93.2% and a specificity of 97.7%. The reasons why the four definitions were missed are provided in Supplementary Data File S4.

### Outcome reporting

#### Distribution of pregnancy outcomes

**Figure 3.**
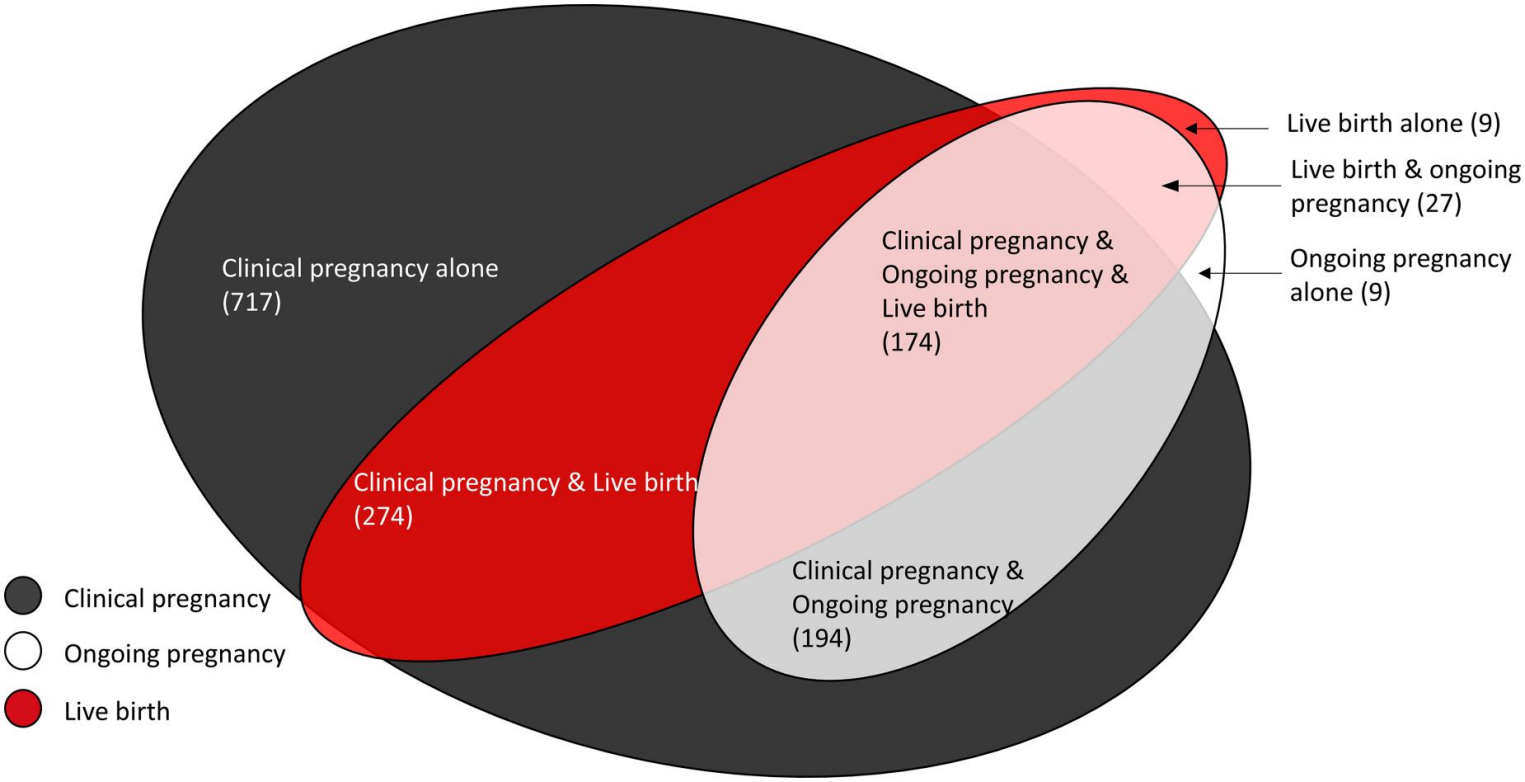
**Number of trials that reported clinical pregnancy, ongoing pregnancy, live birth, and their combinations (n = 1404).** We did not plot 21 studies as they solely reported biochemical pregnancy.

Among the 1425 studies, biochemical pregnancy was reported in 29.4% (n = 419) of studies, while clinical pregnancy, ongoing pregnancy, and live birth were reported in 95.4% (n = 1359), 28.4% (n = 404), and 34.0% (n = 484) of the trials, respectively. Among the 1425 trials, 50.3% of the trials (n = 717) reported clinical pregnancy only, while 12.2% (n = 174) concurrently reported clinical pregnancy, ongoing pregnancy, and live birth (Fig. 3).

#### Comparison of trial characteristics based on reporting endpoints

A comparison of trial characteristics in studies which reported up to biochemical or clinical pregnancy (n = 738), ongoing pregnancy (n = 203), and live birth (n = 484) is presented in Table 1. Among the 1425 trials, those reporting up to biochemical or clinical pregnancy were significantly more likely to be unregistered (62.9%) compared to those reporting up to ongoing pregnancy (38.4%) and live birth (28.7%). Trials reporting up to biochemical or clinical pregnancy randomized fewer patients, with an average of 113 patients, compared to 165 and 179 patients in trials that reported up to ongoing pregnancy and live birth, respectively (*P* < 0.01). Trials reporting up to biochemical pregnancy or clinical pregnancy were more often conducted at a single center (90.8% vs 79.3% vs 70.9%; *P *<* *0.01) and were more likely to report no funding (84.7% vs 76.4% vs 65.3%; *P *<* *0.01). Additionally, they were less likely to be published in first-quarter journals (12.5% vs 35.0% vs 53.5%; *P *<* *0.01) and had fewer citations (7 vs 13 vs 14; *P *<* *0.01).

**Table 1.** Characteristics of trials that reported up to biochemical or clinical pregnancy, ongoing pregnancy, and live birth.

	Report up to biochemical or clinical pregnancy	Report up to ongoing pregnancy	Report up to live birth	*P*-value*
No. of trials	738	203	484	
Trial registration				*P *<* *0.001
Yes	274 (37.1)	125 (61.6)	345 (71.3)	
No	464 (62.9)	78 (38.4)	139 (28.7)	
Sample size, median (IQR)	113 (112)	165 (164)	179 (276)	*P *<* *0.001
Single or multicenter				*P *<* *0.001
Single center	670 (90.8)	161 (79.3)	343 (70.9)	
Multi-center	68 (9.2)	42 (9.2)	141 (29.1)	
Patient recruitment country				*P *<* *0.001
China	173 (23.4)	29 (14.3)	108 (22.3)	
Egypt	85 (11.5)	24 (11.8)	29 (6.0)	
Iran	183 (24.8)	56 (27.6)	45 (9.3)	
European Union	80 (10.8)	49 (24.1)	145 (30)	
Other countries	217 (29.5)	45 (22.2)	157 (32.4)	
Funding bodies				*P *<* *0.001
Industry	13 (1.8)	10 (18.7)	32 (28.1)	
Government	100 (13.6)	38 (4.9)	136 (6.6)	
None	625 (84.6)	155 (76.4)	316 (65.3)	
Year of publication				*P *<* *0.001
2012–2016	244 (33.1)	90 (44.3)	128 (26.4)	
2017–2020	324 (43.9)	75 (36.9)	218 (45.0)	
2021–2023	170 (23.0)	38 (18.7)	138 (28.5)	
First quarter journal or not				*P *<* *0.001
First quarter journal	92 (12.5)	71 (35.0)	259 (53.5)	
Non first quarter journal	646 (87.5)	132 (65.0)	225 (46.5)	
Citation times, median (IQR)	7 (15)	13 (28)	14 (28)	*P *<* *0.001

Data are presented as numbers (percentages) unless otherwise specified.

* The Kruskal–Wallis rank sum test was used for comparing continuous variables and the chi-square test was used for comparing categorical variables. The list of first quarter journals was derived from Clarivate’s Journal Citation Reports in 2023 and citation counts were obtained from Dimensions; EU, European Union.

#### Temporal trend in reporting pregnancy outcomes

Over time, the proportion of the trials reporting up to live birth rose from 23.1% in 2012 to 33.7% in 2023 (Supplementary Fig. S1). During this period, the proportion of trials reporting up to ongoing pregnancy has reduced from 20.8% to 12.0%, while the proportion of trials reporting up to biochemical pregnancy or clinical pregnancy has remained steady.

### Outcome definitions

#### Biochemical pregnancy

Of 419 RCTs that had reported outcomes on biochemical pregnancy, biochemical pregnancy was defined in 68.5% of RCTs (n = 287). Of these 287 definitions, 37.3% of the definitions (n = 107) were based on urinary HCG results whereas 62.7% (n = 180) relied on serum HCG levels. However, 30 out of 287 (10.5%) definitions described that biochemical pregnancy was only established when the HCG rise was transient, equivalent to the definition of biochemical pregnancy loss, while the rest of the 257 definitions described biochemical pregnancy as positive HCG results, without mentioning whether HCG was transient or not. Regarding the time points at biochemical pregnancy confirmation, 59.2% of the 287 definitions (n = 170) had specified when the HCG levels were tested, with 14 days after embryo transfer being the most often used cut-off time point, making up 38.7% (n = 111) of the definitions (Table 2), while 40.8% of the definitions (n = 117) did not mention when biochemical pregnancy was confirmed.

**Table 2.** Frequency of the timeframe used in biochemical pregnancy, clinical pregnancy, ongoing pregnancy, and live birth definitions.

Pregnancy endpoints	Range	No. of definitions	Proportions
**Biochemical pregnancy in days after embryo transfer**	**7–21 days**	**287**	
<14		22	7.7%
14		111	38.7%
>14		37	12.8%
No timepoint used		117	40.8%
**Clinical pregnancy**			
In gestational weeks	4–16 weeks	220	
<6		41	18.6%
6		106	48.2%
7		45	20.5%
>7		28	12.7%
In weeks post-embryo transfer	2–12 weeks	199	
<4		20	10.1%
4		74	37.2%
5		57	28.6%
>5		48	24.1%
In weeks post-positive HCG test	1–8 weeks	108	
<2		5	4.6%
2		52	48.1%
3		17	15.8%
>3		34	31.5%
Since the time of ovulation		28	
No timepoints used		321	
**Ongoing pregnancy**	**6–32 gestational weeks**	**285**	
<10 gestational weeks		23	8.0%
10–11 gestational weeks		37	13.0%
12 gestational weeks		140	49.1%
>12 gestational weeks		52	18.2%
In weeks after embryo transfer		22	7.8%
No timepoints used		11	3.9%
**Live birth**	**20–37 gestational weeks**	**199**	
<22 gestational weeks		17	8.5%
22–23 gestational weeks		17	8.5%
24 gestational weeks		57	28.6%
>24 gestational weeks		33	16.6%
No timepoints used		75	37.7%

#### Clinical pregnancy

**Figure 4.**
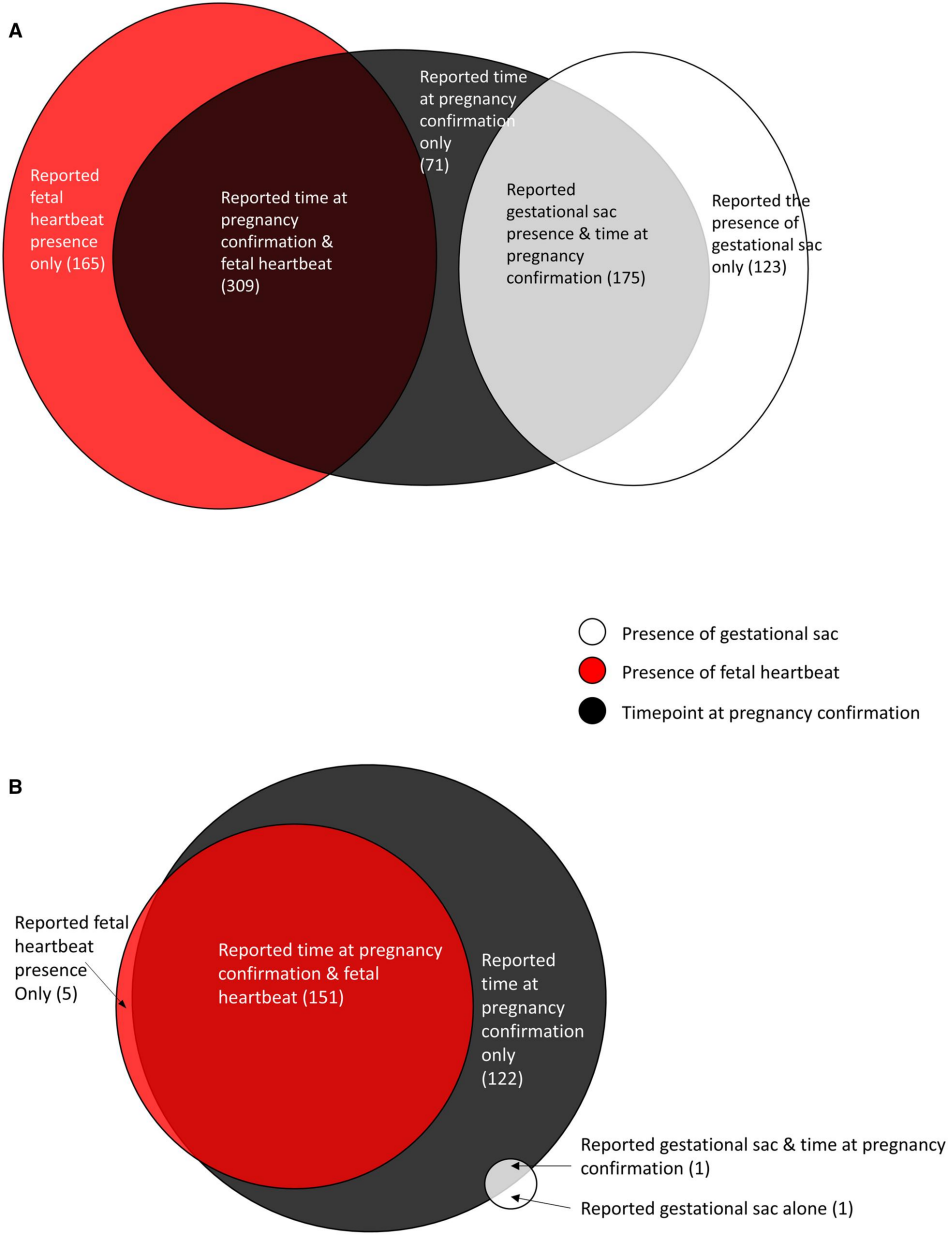
**The number of elements in the clinical pregnancy definitions (n = 843) and ongoing pregnancy definitions (n = 279).** (**A**) clinical pregnancy. Thirty-three definitions were not plotted in clinical pregnancy plot as they did not use the above four components; they used solely HCG levels or whether ultrasound was performed. (**B**) ongoing pregnancy. We did not plot six studies reporting ongoing pregnancy in this graph as they described ongoing pregnancy as ultrasound scanning without mentioning other details.

Of 1359 RCTs that had reported clinical pregnancy, clinical pregnancy was defined in 64.5% of these definitions (n = 876). Various elements were used for determining clinical pregnancy, including the time point at the pregnancy confirmation (n = 555, 63.4%), the presence of the gestational sac(s) (n = 298, 34.0%), and the presence of fetal heartbeat(s) (n = 474, 54.1%) (Fig. 4A). There were 309 (35.3%) studies that defined clinical pregnancy using both time at pregnancy confirmation and the presence of fetal heartbeat. There were 175 (20.0%) definitions that defined clinical pregnancy using both time at pregnancy confirmation and the presence of gestational sac. The time points used for ascertaining clinical pregnancy include gestational weeks, weeks post-embryo transfer, weeks post-positive HCG or weeks after ovulation (Table 2). Among clinical pregnancy definitions that included gestational weeks, they ranged from 4 to 16 gestational weeks. Gestational weeks 6 was the most used time point to establish clinical pregnancy (n = 106), while 36.6% of 876 clinical pregnancy definitions (n = 321) did not use any time points in their definitions.

#### Ongoing pregnancy

Of 404 RCTs that had reported ongoing pregnancy, ongoing pregnancy was defined in 70.5% (n = 285). Of these 285 ongoing pregnancy definitions, time points at the pregnancy confirmation were reported in 96.1% of definitions (n = 274) (Fig. 4B; Table 2), while the presence of fetal heartbeat was described in 54.7% of definitions (n = 156) and the presence of gestational sac only without describing fetal heart beating was mentioned in 0.35% of definitions (n = 1). Among the 285 definitions for ongoing pregnancy, gestational weeks ranged from 6 to 32 weeks, with 140 definitions established at 12 gestational weeks, the most used time point (Table 2).

#### Live birth

Of 484 RCTs that had reported data on live birth, live birth was defined in 41.1% of RCTs (n = 199). Among the 199 live birth definitions, 37.7% (n = 75) did not use any type of time points to describe when the live birth took place, and 28.6% of studies (n = 57) used 24 gestational weeks. The gestational weeks used for establishing live births ranged from 20 to 37 weeks, and 24 weeks was the most used cutoff. There were four studies that required the baby to be alive after birth, including three studies that required at least a week and another study that required the baby to live for over 24 weeks. There were four studies requiring the baby to be healthy, without giving specific criteria to qualify healthy status.

Among 52 trials that reported cumulative live births, only seven trials provided their definitions. Two trials limited the embryo transfer cycles in their definitions to six or three cycles; three trials used per started treatment cycle as part of the definitions; another two specified a 1-year follow-up timeframe of live births after the first embryo transfer. A complete list of definitions is provided in Supplementary Data File S5.

#### Temporal trend in reporting definitions

The proportion of trials reporting definitions in live birth rose sharply from 16.7% in 2012 to 41.9% in 2023 (Supplementary Fig. S2), while the proportion of trials reporting definitions in biochemical pregnancy, clinical pregnancy, and ongoing pregnancy remained stable during this period.

## Discussion

In our analysis of 1425 RCTs in infertility published in the last decade, we found only a third of studies had reported live birth, while concurrently reporting clinical pregnancy, ongoing pregnancy, and live birth accounted for less than one-eighth of all RCTs. Compared to trials that reported up to ongoing pregnancy or live birth, trials that only reported up to biochemical pregnancy or clinical pregnancy tended to be small, single-center, unregistered, and more likely to be published in non-first-quarter journals. Encouragingly, there has been a consistent increase in the proportion of trials that reported live birth over time. While there was a frequent absence of definitions for pregnancy outcomes, there has been an increase in the proportion of trials giving a definition in live birth. However, where the definitions were provided, they often lacked clarity and consistency across trials.

The strength of our study includes a large sample size encompassing a comprehensive collection of infertility trials across a decade, allowing us to have a thorough understanding of the trend in this field. This large sample size enables us to explore reporting patterns over time, a novel aspect that, to the best of our knowledge, has not been investigated before. In addition, we did not restrict our search to English-language articles, enabling us to have a panoramic understanding of the trend across different countries. Lastly, by examining the components and time cut-off criteria used for defining pregnancy outcomes, we provided a more granular picture on the clarity and inconsistency of the reporting.

The reason behind the low uptake of live birth reporting among infertility trials is multifactorial. Compared to live birth, clinical pregnancy or ongoing pregnancy takes a shorter time to follow up, serving as a strong incentive for authors to choose pregnancy over live birth for convenience. In addition, tracking live birth often extends beyond fertility clinics, posing practical challenges in follow-up. Selective reporting may be another cause if results on pregnancy were statistically significant while those on live birth were not. The clustering of reporting of clinical pregnancy and insufficient reporting of live birth tends to make analysis on live birth underpowered, leading to uncertainties in conclusions. To address the issue, professional bodies such as ESHRE or expert opinions have all recommended reporting live birth in infertility trials (Barnhart, 2014; Duffy *et al.*, 2020a, b). This recommendation contributed to the temporal increase in the proportion of trials reporting live birth seen in our results. However, there remains a gulf between the advocated outcome and what researchers are reporting. One way to bridge the gap is to raise awareness of reporting live birth among researchers and improve the uptake of COS. The rationale for not reporting live birth should be provided whenever possible during the stage of study design, trial registration, and manuscript submission. Our findings suggest journals have a powerful role in increasing the reporting of live birth: nearly 90% of trials that reported up to biochemical or clinical pregnancy were published in non-first-quarter journals, compared to around half for trials that reported up to live birth. That being said, live birth is not the sole gold standard measurement for effectiveness in infertility treatments (Wang *et al.*, 2023). Future studies should also explore whether a strong correlation in treatment effects between the early pregnancy stage and live birth could be demonstrated.

Notably, the remarkable differences in trials’ characteristics among those reported to different stages of pregnancy have profound implications for the interpretation of research findings. The common traits of trials reporting up to biochemical or clinical pregnancy include small sample size, single-center study design, and lack of trial registration–these are also ingredients of publication reporting bias, leading to inflated treatment effects compared to what might be observed in larger, multicenter, registered trials. Given that trials reporting up to biochemical or clinical pregnancy accounted for nearly half of all infertility studies, such prevalence underscores their potential to overturn conclusions drawn from many secondary analyses lacking data on live birth.

Our study builds on prior research and provides a more nuanced understanding of reporting patterns in pregnancy definitions, which can be summarized into three key aspects, including (i) widespread absence, (ii) insufficient clarity, and (iii) vast heterogeneity in the definitions (Wilkinson *et al.*, 2016). Regarding the absence of definitions, we confirmed the similar extent of missing definitions observed in smaller-scale studies (Rimmer *et al.*, 2022). Our findings are also consistent with Wilkinson *et al.* (2016), who found that half of 52 studies that reported live births did not define the outcome and a fifth of studies reported clinical pregnancy but did not give definitions. Our work has identified an encouraging trend despite the widespread absence of reporting definitions. An increasing proportion of trials are reporting definitions of live birth, indicating that the research community is recognizing the importance of this issue.

Regarding the insufficient clarity in definitions, there was a pervasive omission of the timing of pregnancy confirmation in the definitions of biochemical pregnancy, clinical pregnancy, and live birth. This is a significant source of confusion for readers and users of RCTs, particularly for those conducting meta-analyses who cannot pin down the accurate pregnancy stages for pooling data. This leads to potential inaccuracies in the synthesized findings and conclusions. However, an exception was ongoing pregnancy, where less than 5% of definitions had missing timing at definition. We speculate the reasons could be that, unlike earlier pregnancy stages that often rely on diagnostic tools such as ultrasound or serum HCG levels, the criteria to establish ongoing pregnancy are more universally recognized as the continuum of pregnancy in time. Another factor could be that trialists need to include timing at confirmation of ongoing pregnancy to differentiate it from clinical pregnancy when both are reported.

This vast heterogeneity in outcomes that are ostensibly identical is also reflected in the components used to define pregnancy. These include HCG levels, the presence of gestational sac only, fetal heartbeat presence, and various gestational weeks used. The definitions are also inconsistent from trial to trial, and some are even erroneous. For example, biochemical pregnancy loss, characterized by transient HCG rise, was sometimes mistakenly defined as biochemical pregnancy.

The reasons behind the frequently absent, unclear, inconsistent, and sometimes erroneous definitions are multifactorial. Some trialists were neither aware of the standardized outcome definitions nor required to use them at trial registration. At the publication stage, few journals or reviewers required authors to provide standardized definitions of outcomes if they were not given. The lack of consistency in pregnancy definitions recommended by different expert opinions further perpetuates the heterogeneity of pregnancy definitions. For example, recommendations on the definition of ongoing pregnancy are absent. The definition of live birth by the World Health Organization does not give precise weeks of gestation (World Health Organization 2024), while live birth is defined as 22 weeks of gestation in ICMART (Zegers-Hochschild *et al.* 2017a, b) and 20 weeks of gestation by the COS in infertility (Duffy *et al.* 2021). Justifications for the inconsistency of gestational weeks provided by the COS were based on advancements in perinatal and neonatal medicine (Supplementary Table S1) (Duffy *et al.* 2021). Nevertheless, this inconsistency has led to the publication of studies that had no clear definitions or used non-standardized definitions, entrenching the misbelief of the readers that such inconsistent reporting is acceptable.

We also observed varying degrees of alignment between the pregnancy definitions provided by trialists and the recommended standards (Legro *et al.*, 2014; World Health Organization 2024). In terms of biochemical pregnancy, all definitions identified in our study align with ICMART’s recommendation in 2017 (Zegers-Hochschild *et al.*, 2017a, b), which defined biochemical pregnancy as the detection of beta HCG in serum or urine. To the best of our knowledge, ICMART is the only recommendation that defines biochemical pregnancy. However, a limitation of this definition is that ICMART did not specify whether the detection of HCG is transient or persistent. Including this temporal aspect is crucial to differentiate between biochemical pregnancy loss and biochemical pregnancy. Regarding clinical pregnancy, the most commonly used definitions consider both the timing of pregnancy confirmation and the presence of a gestational sac, with or without a fetal heartbeat. This is consistent with the definition of clinical pregnancy in the ICMART’s recommendation (Zegers-Hochschild *et al.*, 2017a, b). Surprisingly, despite clinical pregnancy being widely used, it is rarely defined in recommendations, with ICMART being the only organization to provide a definition. While viable intrauterine pregnancy was defined in the core outcomes sets, the gestational weeks were not provided and whether this corresponds to clinical or ongoing pregnancy is unclear to readers. Regarding ongoing pregnancy, a recommended standardized definition does not exist. Based on our observation, the most commonly used definition for ongoing pregnancy is 12 weeks of gestation, with or without describing the presence of a fetal heartbeat. We propose that this definition could be adopted in future recommendations for ongoing pregnancy to improve the consistency of this definition. In terms of live birth, while the most commonly used definition is a gestational age beyond 24 weeks, none of the recommended guidelines align with this widely accepted definition. To standardize the definitions of live birth, future research should explore why the uptake of the recommended definitions of live birth was low.

Our study has limitations. Firstly, despite our systematic search strategy, we missed seven out of 84 articles in our test set. It is possible that some relevant articles were published outside the databases we searched or did not match the criteria of our search strategy. Secondly, our findings only represent the trend in RCTs of those published. There is likely to be a greater proportion of RCTs that remain unpublished, potentially presenting a different picture of outcome reporting and definitions that warrant investigation for future studies. Thirdly, we chose a sample size of 50 articles for manual validation as it was practical, achieved high sensitivity and specificity, and aligned with validation methods commonly used by researchers working with large datasets (Chavalarias *et al.*, 2016; Szucs and Ioannidis, 2017). However, this limited sample may lack the statistical power to fully validate the reliability of our algorithm. Fourthly, while our hybrid data extraction improved the efficiency of our work, it is not infallible, particularly when determining whether an outcome was reported by a study and when pregnancy definitions were given but not successfully extracted. However, this approach was necessary given the vast amount of data we needed to extract and the diverse ways the information was embedded in the text. Our manual checks revealed that these errors occurred in 10% or fewer of the studies. Fifthly, our study reported the presence of a fetal heartbeat as part of the definition of clinical pregnancy, rather than treating it as a distinct outcome measure, as defined in the International Glossary on Infertility and Fertility Care (Zegers-Hochschild *et al.*, 2017a, b). This reflects common reporting practices in the literature but may limit comparability with studies that separate these outcomes. Lastly, we limited our scope to successful pregnancy endpoints, including pregnancy or live birth, and did not evaluate the definitions of miscarriage; incorporating this aspect would provide a more comprehensive understanding of pregnancy outcome reporting.

## Conclusion

Although there is a long-running recommendation to report live birth in infertility trials, only a small fraction of RCTs adhered to the recommendation. This discrepancy underscores a gap between recommended and reported outcomes. Encouragingly, there has been a consistent increase in the proportion of trials reporting live birth. Furthermore, there were significant inconsistencies, lack of clarity, and wide variations in the definitions of pregnancy outcomes, which could distort treatment effects and hinder evidence synthesis. Our work highlights a critical need to increase the uptake of standardized criteria for reporting these outcomes.

## Supplementary Material

deaf022_Supplementary_Data_Files

deaf022_Supplementary_Figure_S1

deaf022_Supplementary_Figure_S2

deaf022_Supplementary_Table_S1

## Data Availability

The data underlying this article will be shared on reasonable request to the corresponding author.
