# Supplementary material for: The presence, clarity, and consistency of definitions in pregnancy outcomes in infertility trials: a systematic review
Source: Hum Reprod. 2025 Feb 21;40(4):654–63. doi: 10.1093/humrep/deaf022 (PMC11965792; doi:10.1093/humrep/deaf022)
Supplement: deaf022_Supplementary_Data_Files [file deaf022_supplementary_data_files.docx]

# Supplementary Data File S1 Search strategy in Medline, Embase and Central (searched on 30 August 2023)

Medline

| 1 | exp reproductive procedure/ |
| --- | --- |
| 2 | exp fertility promoting agent/ |
| 3 | (in vitro adj3 fertili$).tw. |
| 4 | ivf.tw. |
| 5 | icsi.tw. |
| 6 | (intracytoplas$ adj5 sperm$).tw. |
| 7 | reproductive technology.tw. |
| 8 | exp infertility/ |
| 9 | exp fertility/ |
| 10 | exp fertilization/ |
| 11 | exp insemination/ |
| 12 | exp nidation/ |
| 13 | (clomifene citrate or letrozole or polycystic ovarian syndrome or PCOS or inferti$ or gonadotropin).ti,ab. |
| 14 | ((artificial$ or cervi* or intrauterine or intra-uterine) adj3 inseminat$).tw. |
| 15 | (tim$ adj2 ovulat$).tw. |
| 16 | (ovulat$ adj3 (induc$ or stimulat*)).tw. |
| 17 | exp ovulation induction/ |
| 18 | superovulation.tw. |
| 19 | (ovar$ adj2 (stimulat$ or hyperstimula$)).tw. |
| 20 | (control* adj2 ovulat*).tw. |
| 21 | (oocyt$ adj5 (retriev$ or pickup)).tw. |
| 22 | ((embry$ or blastocyst*) adj5 (biops$ or transfer*)).tw. |
| 23 | ((embryo or blastocyst$ or cleavage) adj2 transfer).ti,ab. |
| 24 | (cocult$ adj3 (trophobl$ or embry$)).tw. |
| 25 | exp ovary hyperstimulation/ |
| 26 | ohss.tw. |
| 27 | (luteal adj3 (defect$ or dysfunct$ or support)).tw. |
| 28 | or/1-27 |
| 29 | exp pregnancy/ |
| 30 | exp pregnancy outcome/ |
| 31 | exp pregnancy rate/ |
| 32 | exp live birth/ |
| 33 | exp prepregnancy care/ |
| 34 | (pregnan$ or (implant$ adj2 rate$) or birth$ or fetal$ or miscarriage$ or pregnancy loss$ or abortion$ or fetus$ or foetus or conception$).mp. |
| 35 | or/29-34 |
| 36 | Clinical trial/ |
| 37 | Randomized controlled trial/ |
| 38 | Randomization/ |
| 39 | Single blind procedure/ |
| 40 | Double blind procedure/ |
| 41 | Crossover procedure/ |
| 42 | Placebo/ |
| 43 | Randomi?ed controlled trial$.tw. |
| 44 | Random allocation.tw. |
| 45 | Randomly allocated.tw. |
| 46 | Allocated randomly.tw. |
| 47 | (allocated adj2 random).tw. |
| 48 | (allocated adj2 random).tw. |
| 49 | Single blind$.tw. |
| 50 | Double blind$.tw. |
| 51 | ((treble or triple) adj blind$).tw. |
| 52 | Placebo$.tw. |
| 53 | Prospective study/ |
| 54 | random$.ti,ab. |
| 55 | or/36-54 |
| 56 | Case study/ or systematic review/ or meta-analysis/ or exp "review"/ or Abstract report/ or letter/ |
| 57 | (Case report or meta-analysis or meta analysis or systematic review or study protocol or cost-effectiveness or cost effectiveness or (prospective adj2 cohort) or (retrospective adj2 analy*) or (retrospective adj2 study)).ti. |
| 58 | exp animal experiment/ or exp animal model/ or exp experimental animal/ or exp transgenic animal/ or exp male animal/ or exp female animal/ or exp juvenile animal/ or animal/ or chordata/ or vertebrate/ or tetrapod/ or exp fish/ or amniote/ or exp amphibia/ or mammal/ or exp reptile/ or exp sauropsid/ or therian/ or exp monotreme/ or placental mammal/ or exp marsupial/ or Euarchontoglires/ or exp Afrotheria/ or exp Boreoeutheria/ or exp Laurasiatheria/ or exp Xenarthra/ or primate/ or exp Dermoptera/ or expGlires/ or exp Scandentia/ or Haplorhini/ or exp prosimian/ or simian/ or exp tarsiiform/ or Catarrhini/ or exp Platyrrhini/ or ape/ or exp Cercopithecidae/ or hominid/ or exp hylobatidae/ or exp chimpanzee/ or exp gorilla/ or exp orang utan/ or exp cephalopod/ or (rat or rats or animal or animals or mice or "in vivo" or mouse or rabbit or rabbits or murine or pig or pigs or dog or dogs or bovine or fish or vertebrate or vertebrates or cat or cats or rodent or rodents or mammal or mammals or chicken or chickens or monkey or monkeys or sheep or canine or canines or porcine or cattle or bird or birds or hamster or hamsters or primate or primates or cow or cows or chick or horse or horses or avian or avians or calf or swine or swines).ti,ab,kw. |
| 59 | or/56-58 |
| 60 | 28 and 35 and 55 |
| 61 | 60 not 59 |
| 62 | limit 61 to yr="2012 -Current" |

| Embase | |
| --- | --- |
| 1 | exp reproductive procedure/ |
| 2 | exp fertility promoting agent/ |
| 3 | (in vitro adj3 fertili$).tw. |
| 4 | ivf.tw. |
| 5 | icsi.tw. |
| 6 | (intracytoplas$ adj5 sperm$).tw. |
| 7 | reproductive technology.tw. |
| 8 | exp infertility/ |
| 9 | (clomifene citrate or letrozole or gonadotropin).ti. |
| 10 | exp fertilization/ |
| 11 | exp insemination/ |
| 12 | exp nidation/ |
| 13 | ((artificial$ or cervi* or intrauterine or intra-uterine) adj3 inseminat$).tw. |
| 14 | (tim$ adj2 ovulat$).tw. |
| 15 | (ovulat$ adj3 (induc$ or stimulat*)).tw. |
| 16 | exp ovulation induction/ |
| 17 | superovulation.tw. |
| 18 | (ovar$ adj2 (stimulat$ or hyperstimula$)).tw. |
| 19 | (control* adj2 ovulat*).tw. |
| 20 | (oocyt$ adj5 (retriev$ or pickup)).tw. |
| 21 | ((embry$ or blastocyst*) adj5 (biops$ or transfer*)).tw. |
| 22 | (cocult$ adj3 (trophobl$ or embry$)).tw. |
| 23 | exp ovary hyperstimulation/ |
| 24 | ohss.tw. |
| 25 | (luteal adj3 (defect$ or dysfunct$ or support)).tw. |
| 26 | ((embryo or blastocyst or cleavage) adj2 transfer).ti. |
| 27 | or/1-26 |
| 28 | exp pregnancy/ |
| 29 | exp pregnancy outcome/ |
| 30 | exp pregnancy rate/ |
| 31 | exp live birth/ |
| 32 | exp prepregnancy care/ |
| 33 | exp *pregnancy/ or exp *pregnancy disorder/ or exp *obstetric procedure/ or exp *breast feeding/ or exp *breast feeding education/ or exp *birth/ or exp *childbirth/ or *childbirth education/ or *labor pain/ or (ante natal or antenatal* or pre natal* or prenatal* or puerper* or postnatal* or postpartum or post partum or post natal* or peripartum or peri partum or prepregnancy or pre pregnancy or preconception* or pre conception* or periconception* or peri conception* or ((preterm or premature) and (labor or labour)) or eclamp* or preeclamp* or pre eclamp* or amniocentes* or chorion* vill* or breastfe* or breast fe* or lactation* or cesarean or caesarean or cesarian or caesarian or cesarien or caesarien or newborn* or new born* or tocoly* or fetal or foetal or fetus or foetus or miscarriage* or pregnancy or pregnancies or pregnant).ti,kf. |
| 34 | (pregnan$ or miscarriage$ or pregnancy loss$ or live birth$).ti,ab. |
| 35 | or/28-34 |
| 36 | case study/ |
| 37 | case report.tw. |
| 38 | abstract report/ or letter/ |
| 39 | exp animal/ not human/ |
| 40 | (systematic review or meta analysis or study protocol or prospective cohort or pilot study or protocol paper or narrative review or retrospective or cohort or secondary analysis or cost effective* or sperm or sperms or semen).ti. |
| 41 | (rat or rats or mouse or mice or swine or porcine or murine or sheep or lambs or pigs or piglets or rabbit or rabbits or cat or cats or dog or dogs or cattle or bovine or monkey or monkeys or trout or heifer$ or marmoset$1).ti. |
| 42 | retrospective study/ or review/ |
| 43 | (contracep$ or ectopic pregnanc$ or preterm birth$ or preterm labo$ or pregnancy loss$ or cesarean scar pregnanc$ or ectopic pregnanc$ or trimester or fetal).ti. |
| 44 | (survey or questionnaire or qualitative study or retrospective or editorial or commentary or literature review or review or case control or observational study or good practice or best practice).ti. |
| 45 | (case control or case-control or cross-sectional or cross sectional or case-controlled).ti,ab. |
| 46 | or/36-45 |
| 47 | (Randomized controlled trial/ or Controlled clinical study/ or random$.ti,ab. or randomization/ or intermethod comparison/ or placebo.ti,ab. or (compare or compared or comparison).ti. or ((evaluated or evaluate or evaluating or assessed or assess) and (compare or compared or comparing or comparison)).ab. or (open adj label).ti,ab. or ((double or single or doubly or singly) adj (blind or blinded or blindly)).ti,ab. or double blind procedure/ or parallel group$1.ti,ab. or (crossover or cross over).ti,ab. or ((assign$ or match or matched or allocation) adj5 (alternate or group$1 or intervention$1 or patient$1 or subject$1 or participant$1)).ti,ab. or (assigned or allocated).ti,ab. or (controlled adj7 (study or design or trial)).ti,ab. or (volunteer or volunteers).ti,ab. or human experiment/ or trial.ti.) not (((random$ adj sampl$ adj7 ("cross section$" or questionnaire$1 or survey$ or database$1)).ti,ab. not (comparative study/ or controlled study/ or randomi?ed controlled.ti,ab. or randomly assigned.ti,ab.)) or (Cross-sectional study/ not (randomized controlled trial/ or controlled clinical study/ or controlled study/ or randomi?ed controlled.ti,ab. or control group$1.ti,ab.)) or (((case adj control$) and random$) not randomi?ed controlled).ti,ab. or (Systematic review not (trial or study)).ti. or (nonrandom$ not random$).ti,ab. or "Random field$".ti,ab. or (random cluster adj3 sampl$).ti,ab. or ((review.ab. and review.pt.) not trial.ti.) or ("we searched".ab. and (review.ti. or review.pt.)) or "update review".ab. or (databases adj4 searched).ab. or ((rat or rats or mouse or mice or swine or porcine or murine or sheep or lambs or pigs or piglets or rabbit or rabbits or cat or cats or dog or dogs or cattle or bovine or monkey or monkeys or trout or marmoset$1).ti. and animal experiment/) or (Animal experiment/ not (human experiment/ or human/))) |
| 48 | 27 and 35 and 47 |
| 49 | 48 not 46 |
| 50 | limit 49 to english |
| 51 | limit 50 to article |
| 52 | limit 51 to journal |
| 53 | limit 52 to yr="2012 -Current" |
| 54 | limit 53 to male |
| 55 | 53 not 54 |

| Cochrane Central Register of Controlled Trials | |
| --- | --- |
| 1 | exp fertilization in vitro/ |
| 2 | exp fertilization in vitro/ |
| 3 | exp intracytoplasmic sperm injection/ |
| 4 | in vitro fertili$.tw. |
| 5 | ivf.tw. |
| 6 | icsi.tw. |
| 7 | (intracytoplas$ adj5 sperm$).tw. |
| 8 | assisted reproductive technology.tw. |
| 9 | exp female infertility/ |
| 10 | exp female fertility/ |
| 11 | exp fertilization/ |
| 12 | exp fertilization/ |
| 13 | exp insemination/ |
| 14 | exp nidation/ |
| 15 | ((artificial$ or cervi* or intrauterine or intra-uterine) adj3 inseminat$).tw. |
| 16 | (ovulat$ adj3 (induc$ or stimulat*)).tw. |
| 17 | exp ovulation induction/ |
| 18 | superovulation.tw. |
| 19 | (ovar$ adj2 (stimulat$ or hyperstimula$)).tw. |
| 20 | (control* adj2 ovulation).tw. |
| 21 | (tim$ adj2 ovulat$).tw. |
| 22 | (oocyt$ adj5 (retriev$ or pickup)).tw. |
| 23 | ((embry$ or blastocyst*) adj5 (biops$ or transfer*)).tw. |
| 24 | (cocult$ adj3 (trophobl$ or embry$)).tw. |
| 25 | exp Ovarian Hyperstimulation Syndrome/ |
| 26 | ohss.tw. |
| 27 | (luteal adj3 (defect$ or dysfunct$ or support)).tw. |
| 28 | (letrozole or clomiphene citrate or infertil* or assisted reproduct*).ti. |
| 29 | or/2-28 |
| 30 | exp Pregnancy/ |
| 31 | exp pregnancy outcome/ |
| 32 | exp pregnancy rate/ |
| 33 | exp live birth/ |
| 34 | (pregnan$ or (implant$ adj2 rate$) or birth$ or miscarriage$ or pregnancy loss$ or abortion$ or conception$).ti,ab. |
| 35 | or/30-34 |
| 36 | 35 and 29 |
| 37 | (Trial registry record or Conference proceeding).pt. |
| 38 | study protocol.ti. |
| 39 | or/37-38 |
| 40 | 36 not 39 |
| 41 | limit 40 to yr="2012 -Current" |

# Supplementary Data File S2 The method for developing a test set

Step 1. To develop the test sets, we searched for the Cochrane reviews in infertility published from 1 January 2012 to 10 November 2023 using the following search strategy. We found 339 Cochrane reviews using the following search strategy

Inferti* or subfertil* or vitro fertili*ation or IVF or assisted reproduct* or intrauterine insemination or letrozole or clomiphene citrate or ovarian stimulation or ovulation induction or icsi or intracytoplas* sperm injection or superovulation or ovarian hyperstimulation or OHSS or luteal support or blastocyst or cleavage embryo or embryo transfer or oocyte pick* or oocyte retrieval or gonadotropin or embryo biopsy or frozen embryo transfer or FET or ovarian near/2 stimulation or implantation failure or embryo culture or embryo biops* in Cochrane CENTRAL webpage.

Step 2. Then we manually screened reviews that had a forest plot in reporting live birth, and 78 reviews were included. The DOI list of those 78 reviews are as follows.

10.1002/14651858.CD013063.pub2, 10.1002/14651858.CD013497.pub2, 10.1002/14651858.CD007876.pub2, 10.1002/14651858.CD011320.pub4, 10.1002/14651858.CD013233.pub2, 10.1002/14651858.CD007421.pub4, 10.1002/14651858.CD009517.pub4, 10.1002/14651858.CD003414.pub3, 10.1002/14651858.CD004378.pub3, 10.1002/14651858.CD004752.pub2, 10.1002/14651858.CD011537.pub3, 10.1002/14651858.CD005356.pub3, 10.1002/14651858.CD008528.pub3, 10.1002/14651858.CD005291.pub3, 10.1002/14651858.CD006359.pub3, 10.1002/14651858.CD006109.pub3, 10.1002/14651858.CD005070.pub3, 10.1002/14651858.CD004634.pub4, 10.1002/14651858.CD012692.pub2, 10.1002/14651858.CD001894.pub6, 10.1002/14651858.CD011184.pub3, 10.1002/14651858.CD009577.pub3, 10.1002/14651858.CD000317.pub4, 10.1002/14651858.CD008720.pub2, 10.1002/14651858.CD011809.pub2, 10.1002/14651858.CD011424.pub4, 10.1002/14651858.CD002808.pub3, 10.1002/14651858.CD006105.pub4, 10.1002/14651858.CD012396.pub2, 10.1002/14651858.CD003416.pub5, 10.1002/14651858.CD000099.pub4, 10.1002/14651858.CD003357.pub5, 10.1002/14651858.CD006919.pub4, 10.1002/14651858.CD012693.pub2, 10.1002/14651858.CD006942.pub3, 10.1002/14651858.CD003854.pub2, 10.1002/14651858.CD002811.pub4, 10.1002/14651858.CD003719.pub4, 10.1002/14651858.CD001750.pub4, 10.1002/14651858.CD008046.pub4, 10.1002/14651858.CD010290.pub3, 10.1002/14651858.CD006920.pub3, 10.1002/14651858.CD004829.pub4, 10.1002/14651858.CD009154.pub3, 10.1002/14651858.CD010042.pub2, 10.1002/14651858.CD006900.pub3, 10.1002/14651858.CD013240.pub2, 10.1002/14651858.CD010550.pub2, 10.1002/14651858.CD002249.pub5, 10.1002/14651858.CD009090.pub2, 10.1002/14651858.CD012375.pub2, 10.1002/14651858.CD007689.pub4, 10.1002/14651858.CD002125.pub4, 10.1002/14651858.CD006107.pub4, 10.1002/14651858.CD001122.pub5, 10.1002/14651858.CD001301.pub2, 10.1002/14651858.CD009452.pub2, 10.1002/14651858.CD004832.pub4, 10.1002/14651858.CD012856.pub2, 10.1002/14651858.CD010287.pub4, 10.1002/14651858.CD005996.pub4, 10.1002/14651858.CD001838.pub6, 10.1002/14651858.CD010461.pub3, 10.1002/14651858.CD012378.pub2, 10.1002/14651858.CD001502.pub4, 10.1002/14651858.CD011009.pub2, 10.1002/14651858.CD013505, 10.1002/14651858.CD003053.pub6, 10.1002/14651858.CD009749.pub2, 10.1002/14651858.CD011872.pub3, 10.1002/14651858.CD008189.pub3, 10.1002/14651858.CD010001.pub3, 10.1002/14651858.CD012650.pub2, 10.1002/14651858.CD007807.pub4, 10.1002/14651858.CD003718.pub5, 10.1002/14651858.CD003857.pub4, 10.1002/14651858.CD011345.pub3

Step 3. We manually identified the infertility trials included by the above reviews in Step 3 that had reported live births or ongoing pregnancies, which resulted in 84 infertility trials involving females and had reported live births or ongoing pregnancies. Here are the DOIs of the test sets (n=84).

10.1007/s10815-014-0385-y, 10.1016/j.fertnstert.2012.12.043, 10.1093/humrep/dex231, 10.1007/s00404-014-3541-9, 10.1093/humrep/dew156, 10.1093/humrep/dey334, 10.1002/ijgo.12355, 10.1016/j.rbmo.2017.04.004, 10.1093/humrep/dev224, 10.1159/000363235, 10.1186/s12958-015-0069-1, 10.1093/humrep/dey268, 10.1056/NEJMoa1414827, 10.1016/j.ejogrb.2015.03.023, 10.1093/humrep/dev062, 10.1016/j.ejogrb.2013.03.002, 10.1093/humrep/dey262, 10.1007/s10815-016-0736-y, 10.1093/humrep/dew120, 10.1111/jog.13802, 10.1186/1477-7827-11-96, 10.1093/humrep/dev038, 10.1016/j.ejogrb.2020.03.003, 10.1111/1471-0528.14629, 10.1111/ajo.12168, 10.1016/S0140-6736(17)32406-6, 10.1016/j.fertnstert.2014.03.012, 10.1007/s00404-017-4604-5, 10.1016/j.repbio.2017.05.003, 10.1177/1933719116641764, 10.1056/NEJMoa1513873, 10.1016/j.fertnstert.2016.12.022, 10.1056/NEJMoa1705334, 10.1136/bmj.m2519, 10.1056/NEJMoa1703768, 10.1016/S0140-6736(18)32843-5, 10.1002/uog.14669, 10.1186/s43043-019-0001-2, 10.1007/s10815-017-0949-8, 10.1016/j.mefs.2018.05.002, 10.1177/1933719115602776, 10.1016/j.mefs.2016.06.006, 10.1111/cen.12294, 10.1093/humrep/dew268, 10.1016/j.fertnstert.2016.07.1096, 10.3109/09513590.2013.859242, 10.1016/j.rbmo.2015.04.013, 10.1071/RD13412, 10.1136/bmj.g7771, 10.1016/j.fertnstert.2013.09.035, 10.1016/j.ejogrb.2016.05.027, 10.1007/s40618-013-0021-1, 10.1016/j.ejogrb.2012.11.026, 10.1016/j.fertnstert.2013.09.010, 10.1016/j.fertnstert.2012.12.052, 10.3109/09513590.2013.813475, 10.1093/humrep/dew148, 10.1016/j.mefs.2013.05.012, 10.1111/j.1447-0756.2012.02072.x, 10.1093/humrep/deu263, 10.1001/jama.2017.7217, 10.1093/humrep/dew050, 10.1016/j.jmig.2018.10.013, 10.3892/etm.2015.2690, 10.14660/2385-0868-88, 10.1007/s00404-014-3397-z, 10.1016/S0140-6736(16)00231-2, 10.1093/humrep/dex227, 10.1056/NEJMoa1313517, 10.1080/09513590.2017.1332174, 10.1016/j.fertnstert.2016.05.022, 10.1016/S0140-6736(18)32989-1, 10.3109/09513590.2012.743020, 10.3109/09513590.2015.1101444, 10.1093/humrep/det108, 10.1186/s12958-015-0014-3, 10.4103/0974-1208.117174, 10.1056/NEJMoa1505297, 10.4137/CMRH.S14681, 10.3389/fendo.2018.00545, 10.1186/s12958-018-0343-0, 10.1056/NEJMoa1612337, 10.1142/S2661318219500063, 10.4103/jhrs.JHRS_134_16

Step 4. We compared the list of DOIs in the test set with the RCTs included in our analysis. Among these 84 articles in the test set, the following 7 were not identified in our database.

| DOI | Reasons for why it was incorrectly excluded from our study |
| --- | --- |
| 10.1016/j.mefs.2018.05. | The author stated the design was ‘randomized case control study’ in the abstract, whereas it should be randomized controlled study. |
| 10.3109/09513590.2012.743020 | The author stated the design was ‘randomized case control study’ in the abstract, whereas it should be randomized controlled study. |
| 10.1142/S2661318219500063 | The journal was not indexed in Pubmed or Embase, the two major databases that we searched. |
| 10.4137/CMRH.S14681 | Manual error. It was accidently excluded by the reviewers at screening stage. |
| 10.4103/jhrs.JHRS_134_16 | No keywords such as biochemical/clinical/ongoing pregnancy were reported in the abstract. Therefore, it was incorrectly filtered out at screening stage. |
| 10.14660/2385-0868-88 | The journal was not indexed in Pubmed or Embase. |
| 10.1071/RD13412 | No key words such as IVF/infertile appear in the abstract. Hence it was excluded. |

# Supplementary Data File S3 The number of items incorrectly extracted by automated data extraction among 50 manually checked articles

| **Data name** | **Number of data item incorrectly extracted** | **Details for inaccuracies** |
| --- | --- | --- |
| Recruitment country | 1 | Ascertained incorrect country of origin. |
| Sample size | 4 | Extracted incorrect number of patients being randomized (per protocol analysis) |
| Single or multicenter study | 0 |  |
| Funding | 4 | All four were false negatives, meaning the trial received funding but the data extraction indicates otherwise |
| Trial registration | 2 | Both were false negatives, meaning the trial had been registered but data extraction indicates otherwise |

# Supplementary Data File S4 A breakdown of pregnancy reporting at different stages at manual checking

Among 50 randomly selected articles, manual check results found 47 reported clinical pregnancy, 12 reported ongoing pregnancy and 15 reported live birth. 5 false negative were made in ascertaining clinical pregnancy reporting, 1 false negative was made in ascertaining ongoing pregnancy, 2 false negative and 1 false positives were made in ascertaining reporting of live birth.

|  |  | False positive | False negative |
| --- | --- | --- | --- |
| Clinical pregnancy | 47 | 0 | 5 |
| Ongoing pregnancy | 12 | 0 | 1 |
| Live birth | 15 | 1 | 2 |

The four missed definitions in the 50 randomly selected articles are provided below.

|  | Type of error | Pregnancy definitions | Definition provided in the trials’ report |
| --- | --- | --- | --- |
| 1 | False positive (this was definition of spontaneous abortion but was extracted as clinical pregnancy) | Clinical pregnancy | Spontaneous abortion is defined as the loss of clinical pregnancy before completed 22 weeks’ gestation |
| 2 | False negative | Biochemical pregnancy | The pregnancy rate was determined biochemically (i.e. about 2 weeks after oocyte retrieval) |
| 3 | False negative | Live birth | Followed by confirmation of biochemical pregnancy, clinical pregnancy was approved by detection of fetal heart rate in the 7th week after last menstrual period and live birth rate by physician report. |
| 4 | False negative | Clinical pregnancy | Indications of pregnancy: positive pregnancy test or ultrasound monitoring showing embryo sac, embryo or fetal heartbeat (written in Chinese) |

# Supplementary Data File S5 The list of definitions in cumulative live birth

1. Cumulative live birth rate after six embryo transfer cycles

2. Cumulative live birth rate per started treatment cycle, including the transfer of frozen and thawed embryos.

3. The cumulative LBR after 3 cycles

4. The cumulative live birth rate per started cycle

5. The cumulative live birth rate was calculated as the percentage of women starting stimulation who had one live born neonate

6. The proportion of participants with clinical pregnancy or live birth after 1 year of follow-up

7. The rate of cumulative live birth within 12 months after the first embryo transfer
