# Supplementary material for: The presence, clarity, and consistency of definitions in pregnancy outcomes in infertility trials: a systematic review
Source: Hum Reprod. 2025 Feb 21;40(4):654–63. doi: 10.1093/humrep/deaf022 (PMC11965792; doi:10.1093/humrep/deaf022)
Supplement: deaf022_Supplementary_Figure_S1 [file deaf022_supplementary_figure_s1.pdf]

OXFORD
human reproduction
Human Reproduction, 2025, 1–1
https://doi.org/10.1093/humrep/deaf022
Supplementary Data

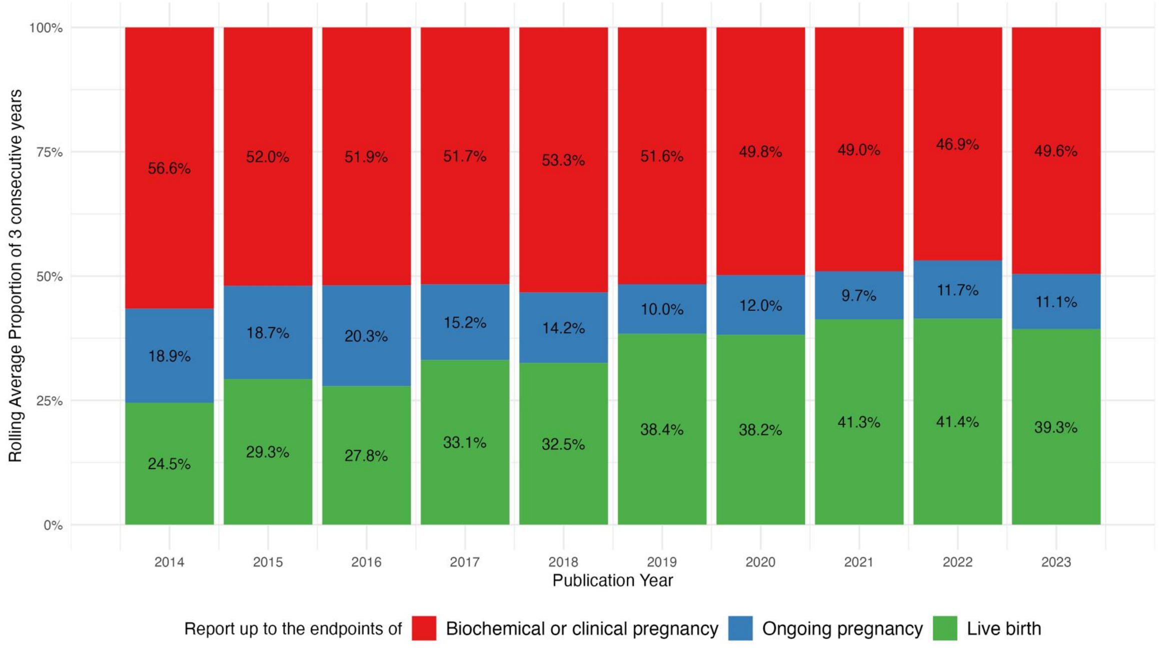

Supplementary Figure S1. The distribution of trials reporting up to biochemical pregnancy or clinical pregnancy, ongoing pregnancy, and live birth, 2014–2023. The data are presented as the rolling average of three consecutive years (e.g. 2012–2014, 2013–2015, etc.).
