# Supplementary material for: The presence, clarity, and consistency of definitions in pregnancy outcomes in infertility trials: a systematic review
Source: Hum Reprod. 2025 Feb 21;40(4):654–63. doi: 10.1093/humrep/deaf022 (PMC11965792; doi:10.1093/humrep/deaf022)
Supplement: deaf022_Supplementary_Figure_S2 [file deaf022_supplementary_figure_s2.pdf]

OXFORD
human reproduction
Human Reproduction, 2025, 1–1
https://doi.org/10.1093/humrep/deaf022
Supplementary Data

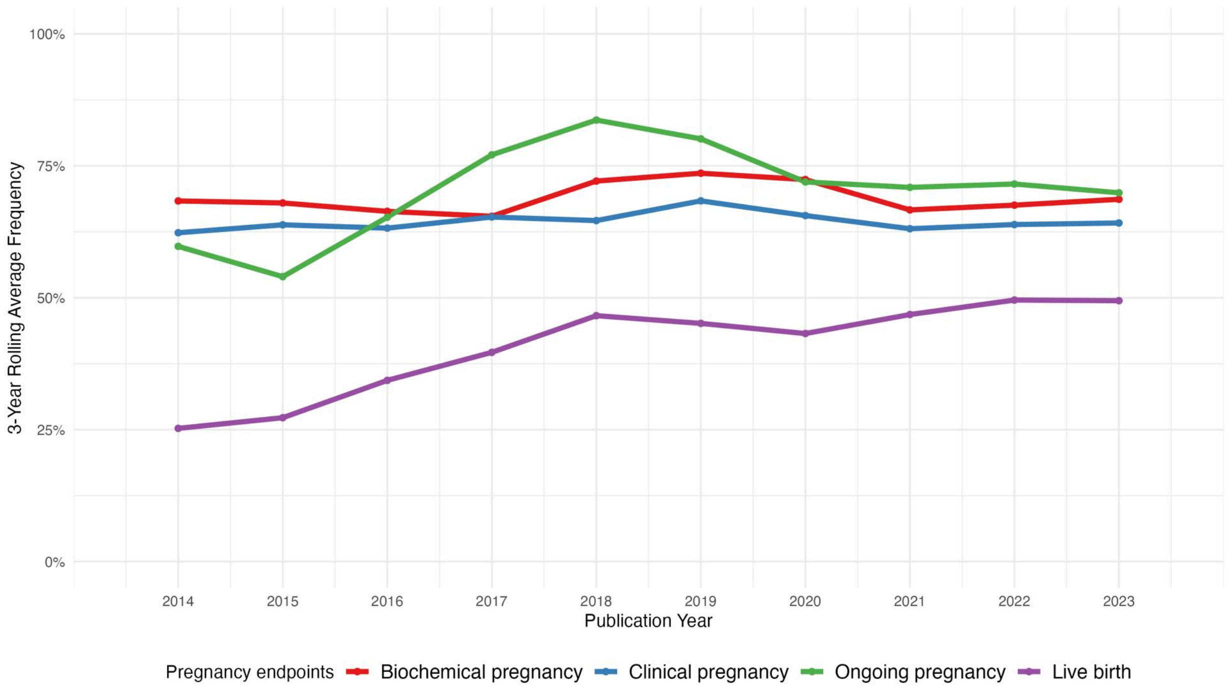

Supplementary Figure S2. The proportion of trials providing definitions in biochemical pregnancy, clinical pregnancy, ongoing pregnancy, or live birth, 2014–2023. The data are presented as the rolling average of three consecutive years (e.g. 2012–2014, 2013–2015, etc.).
