# Supplementary material for: The presence, clarity, and consistency of definitions in pregnancy outcomes in infertility trials: a systematic review
Source: Hum Reprod. 2025 Feb 21;40(4):654–63. doi: 10.1093/humrep/deaf022 (PMC11965792; doi:10.1093/humrep/deaf022)
Supplement: deaf022_Supplementary_Table_S1 [file deaf022_supplementary_table_s1.pdf]

Human Reproduction, 2025, 1–1
https://doi.org/10.1093/humrep/deaf022
Supplementary Data

Supplementary Table S1. Overview of recommended pregnancy or live birth definitions by medical societies and expert consensus.

**The International Glossary on Infertility and Fertility Care (Zegers-Hochschild *et al.* 2017a)**	
Biochemical pregnancy	A pregnancy diagnosed only by the detection of beta hCG in serum or urine
Clinical pregnancy	A pregnancy diagnosed by ultrasonographic visualization of one or more gestational sacs or definitive clinical signs of pregnancy. In addition to intra-uterine pregnancy, it includes a clinically documented ectopic pregnancy.
Clinical pregnancy with heartbeat	A pregnancy diagnosed by ultrasonographic or clinical documentation of at least one fetus with a discernible heartbeat.
Live birth	The complete expulsion or extraction from a woman of a product of fertilization, after 22 completed weeks of gestational age; which, after such separation, breathes or shows any other evidence of life, such as heart beat, umbilical cord pulsation or definite movement of voluntary muscles, irrespective of whether the umbilical cord has been cut or the placenta is attached. A birth weight of 500 g or more can be used if gestational age is unknown. Live birth refer to an individual newborn; for example, a twin delivery represents two live births.
**Improving the Reporting of Clinical Trials of Infertility Treatments (IMPRINT): modifying the CONSORT statement (Harbin Consensus Conference Workshop Group *et al.* 2014)**	
Live birth	Defined as any delivery of a live infant ≥20 weeks gestation
Serum pregnancy	Reported but not defined
Ongoing pregnancy	≥12 weeks
**Standardizing definitions and reporting guidelines for the infertility core outcome set: an international consensus development study (Duffy *et al.* 2021)**	
Viable intrauterine pregnancy confirmed by ultrasound	A pregnancy diagnosed by ultrasonographic examination of at least one fetus with a discernible heartbeat.
Live birth	The complete expulsion or extraction from a woman of a product of fertilization, after 20 completed weeks of gestational age; which, after such separation, breathes or shows any other evidence of life, such as heart beat, umbilical cord pulsation or definite movement of voluntary muscles, irrespective of whether the umbilical cord has been cut or the placenta is attached. A birth weight of 350 g or more can be used if gestational age is unknown.
**World Health Organization**	
Live birth	Live birth is the complete expulsion or extraction from a woman of a fetus, irrespective of the duration of the pregnancy, which, after such separation, shows signs of life.

Zegers-Hochschild F, Adamson GD, Dyer S, *et al.* The International Glossary on Infertility and Fertility Care, 2017. *Fertil Steril*. 2017; 108(3):393-406. doi:10.1016/j.fertnstert.2017.06.005.
Harbin Consensus Conference Workshop Group; Conference Chairs, Legro RS, *et al.* Improving the reporting of clinical trials of infertility treatments (IMPRINT): modifying the CONSORT statement. *Hum Reprod*. 2014; 29(10):2075–2082. doi: 10.1093/humrep/deu218.
Duffy JMN, Bhattacharya S, Bhattacharya S, *et al.* Standardizing definitions and reporting guidelines for the infertility core outcome set: an international consensus development study. *Fertil Steril*. 2021; 115(1):201-212. doi:10.1016/j.fertnstert.2020.11.013.
World Health Organization. *Definition of Live Birth*. https://icd.who.int/dev11/l-m/en#/http://id.who.int/icd/entity/523071804 (12 June 2024, date last accessed).

© The Author(s) 2025. Published by Oxford University Press on behalf of European Society of Human Reproduction and Embryology.
This is an Open Access article distributed under the terms of the Creative Commons Attribution License (https://creativecommons.org/licenses/by/4.0/), which permits unrestricted reuse, distribution, and reproduction in any medium, provided the original work is properly cited.
Human Reproduction, 2025, 00, 1–13
https://doi.org/10.1093/humrep/deaf022
Review
